# Cloning, purification and characterization of trehalose-6-phosphate synthase from *Pleurotus tuoliensis*

**DOI:** 10.7717/peerj.5230

**Published:** 2018-07-12

**Authors:** Xiangli Wu, Zhihao Hou, Chenyang Huang, Qiang Chen, Wei Gao, Jinxia Zhang

**Affiliations:** 1 Institute of Agricultural Resources and Regional Planning, Chinese Academy of Agricultural Sciences, Beijing, China; 2 Key Laboratory of Microbial Resources, Ministry of Agriculture, Beijing, China

**Keywords:** Expression, Trehalose-6-phosphate synthase, Cloning, Purification, *Pleurotus tuoliensis*, Characterization

## Abstract

*Pleurotus tuoliensis*, a kind of valuable and favorable edible mushroom in China, is always subjected to high environmental temperature during cultivation. In our previous study with *P. tuoliensis*, trehalose proved to be effective for tolerating heat stress. Trehalose-6-phosphate synthase (TPS; EC2.4.1.15) plays a key role in the biosynthesis of trehalose in fungi. In this study, a full-length of cDNA with 1,665 nucleotides encoding *TPS* (*PtTPS*) in *P. tuoliensis* was cloned. The PtTPS amino acid was aligned with other homologues and several highly conserved regions were analyzed. Thus, the TPS protein was expressed in *Escherichia coli* and purified by affinity chromatography to test its biochemical properties. The molecular mass of the enzyme is about 60 kDa and the optimum reaction temperature and pH is 30 °C and 7, respectively. The UDP-glucose and glucose-6-phosphate were the optimum substrates among all the tested glucosyl donors and acceptors. Metal cations like Mg^2+^, Co^2+^, Mn^2+^, Ni^2+^, K^+^, Ag^+^ stimulated PtTPS activity significantly. Metal chelators such as sodium citrate, citric acid, EDTA, EGTA and CDTA inhibited enzyme activity. Polyanions like heparin and chondroitin sulfate were shown to stimulate TPS activity.

## Introduction

*Pleurotus tuoliensis* (previously named as *P. eryngii* var. *tuoliensis*) (Zhao et al., 2016) is a favored edible mushroom with rich nutritional and medicinal value in China. It is seasonally cultivated in agricultural facilities in some parts of China, and the spontaneous high environmental temperatures during summer usually affect its growth, development, and finally, the quality and yield of the fruiting body.

Trehalose is a stable and non-reducing disaccharide, which is widely spread in a variety of organisms (Elbein et al., 2003). Trehalose in vivo always acts as an energy source (Paul et al., 2008) and a protectant against various stresses such as heat shock, desiccation, cold, and oxidation (Elbein et al., 2003). At least five trehalose biosynthetic pathways have been reported in the organisms (Avonce et al., 2006). The best characterized and the most widely distributed pathway comprised of two enzymatic steps involving two enzymes, trehalose-6-phosphate synthase (TPS) and trehalose-6-phosphate phosphatase (TPP). In the first step, TPS transfers the glucose from UDP-glucose (UDPG) to glucose-6-phosphate (G-6-P) to form trehalose-6-phosphase which is dephosphorylated by TPP to produce free trehalose in the second step (Avonce et al., 2006; Elbein et al., 2003; Svanström et al., 2014).

Heat stress induced in vivo accumulation of trehalose in many organisms such as *Rhizopus oryzae* (Uyar, Hamamci & Turkel, 2010), *Aspergillus fumigatus* (Al-Bader et al., 2010) and *Saccharomyces cerevisiae* (Avonce et al., 2006; De Virgilio et al., 1994), etc. Disruption of *TPS* gene increased cell sensitivity to heat (Doehlemann, Berndt & Hahn, 2006; Reina-Bueno et al., 2012) and over-expression of *TPS* gene resulted in the toleration of heat stress (Miranda et al., 2007; Soto et al., 1999). Thus, trehalose was previously regarded to serve as a protector against heat stress. But recently, some researchers found in *S. cerevisiae* that not trehalose but TPS protein protected cells from various stresses including heat stress (Gibney et al., 2015; Petitjean et al., 2015). The TPS protein maintained ATP levels to keep energy homeostasis, which is crucial for stress resistance (Petitjean et al., 2015).

Our group has previously studied the effects of trehalose on *P. tuoliensis* during heat stress (Kong et al., 2012). The expression of partial *TPS* gene was elevated and the trehalose content accumulated when *P. tuoliensis* mycelia were exposed to heat stress. Exogenous addition of trehalose significantly reduced the oxidative damages resulted from heat stress to the cell membrane. These results indicated that both the trehalose and the *TPS* gene in *P. tuoliensis* were important in tolerating heat stress.

In this report, a complete *TPS* cDNA was cloned successfully by Rapid Amplification of cDNA Ends (RACE)-PCR and the deduced amino acids were aligned with the other homologues from several organisms. The response of mycelial TPS to the heat stress was studied by detecting its expression quantities through real-time fluorescent quantitative PCR (qPCR) and TPS enzyme activities. The TPS protein was also expressed in *Escherichia coli* and purified to test its biochemical properties. The results will be useful for understanding the role of the TPS gene and the enzyme from *P. tuoliensis*.

## Materials and Methods

### Strains, culture conditions, plasmids, and chemicals

*Pleurotus tuoliensis* (CCMSSC 00489) was provided by the China Center for Mushroom Spawn Standards and Control (CCMSSC). The mycelia were grown on potato-dextrose agar (PDA) at 25 °C for 10 days and were then used for total RNA extraction. BL21 (DE3) and DH5a competent cells were bought from Tiangen Biotech CO., Ltd. (Beijing, China). Transformation of the plasmids to the former two strains was conducted as the provider suggested. All the restriction endonucleases were purchased from NEB Ltd. (Beijing, China). The pGEM^®^-T Easy Vector used for cloning PCR products was bought from Promega Biotech Co., Ltd. (Beijing, China). All the chemicals in the study were purchased from Sigma-Aldrich Co., Ltd. (Shanghai, China) and Sangon Biotech Co., Ltd. (Shanghai, China).

### RNA extraction and cDNA synthesis

Total RNA was extracted from *P. tuoliensis* mycelia using RNAprep Pure Plant Kit (Tiangen Biotech CO., Ltd. (Beijing, China)) which contains DNAase I to digest the genomic DNA. The first strand cDNA was prepared using the M-MuLV (Moloney Murine Leukemia Virus) first strand cDNA synthesis kit (Sangon Biotech Co., Ltd. (Shanghai, China)) and stored at −20 °C for later use.

### Cloning of full length of *TPS* cDNA and sequence analysis

All the primers used in this study were listed in Table S1. The degenerate primers TPS_F1 and TPS_R1 were designed using CODEHOP strategy (http://blocks.fhcrc.org/codehop.html) based on the conserved sequences of TPS from multiple sequence alignments. The total cDNA was used as the PCR template and the PCR reaction solutions in a total volume of 25 μl contained one μl of each degenerate primer (20 μM), one μl of cDNA (30 ng/μl), 0.2 μl of Ex Taq DNA polymerase (TaKaRa), 2.5 μl of dNTP mixture, 2.5 μl of Ex Taq buffer (10×), and 16.8 μl of ddH_2_O. Touchdown PCR was conducted as follows: pre-denaturation at 94 °C for 4 min, 35 cycles of denaturation at 94 °C for 30 s, annealing at 65–60.5 °C (with the decrements of 0.5 °C from 65 to 60.5 °C at each cycle) in the first 10 cycles while at 58 °C in the rest 25 cycles for 30 s, extension at 72 °C for 45 s, and a final extension at 72 °C for 10 min. The obtained PCR product was purified using TIANquick Mini Purification Kit (Tiangen Biotech CO., Ltd. (Beijing, China)) to eliminate the ions and primers. Then the purified PCR product was ligated to pGEM^®^-T Easy Vector as the kit suggested (pGEM^®^-T Easy Vector Systems; Promega Biotech Co., Ltd. (Beijing, China)) to do blue–white selection, and then four white colonies were picked for sequencing (BGI (Beijing, China)). The obtained four sequences were aligned with the homologous *TPS* genes, respectively, and the sequence with the highest identities was considered as a part of *TPS* gene. RACE was conducted as the instructions in the SMARTer^®^ RACE 5′/3′ Kit (Clontech Laboratories, Inc., Mountain View, CA, USA) to get the *TPS* gene fragments at both 5′ and 3′ ends. The gene specific primers and nested primers were designed based on the partial *TPS* sequence obtained through the aforementioned sequencing. The primers 5′RACE-TPS-GSP and 5′RACE-TPS-NGSP were used to clone the 5′ end while the primers 3′RACE-TPS-GSP and 3′RACE-TPS-NGSP were for the 3′ end. The PCR products were purified and ligated into pGEM^®^-T Easy Vector for sequencing (BGI (Beijing, China)), respectively. A pair of primers, TPS-F2 and TPS-R2, was designed based on the sequences at 5′ and 3′ ends and the PCR was carried out to amplify the full cDNA fragment of *TPS*. The *TPS* coding sequences were obtained through the sequencing of the PCR products. The amino acid sequence was deduced by the Expasy tool (http://web.expasy.org/translate/). Multiple homologous amino acid sequences were aligned by the ClustalX program, implemented with Genedoc software and manually edited.

### Heat treatment of *P. tuoliensis* mycelia

*Pleurotus tuoliensis* mycelia were punched at the margin of the growing colonies on PDA and 10 pieces of mycelia discs (five mm in diameter) were inoculated to 100 ml of potato/dextrose medium and shaken at 140 rpm and 25 °C for six days. Then the obtained liquid culture were incubated at 37 °C and treated for 0, 6, 12, 24, and 48 h, respectively. The control was incubated at 25 °C and treated for the same time. All the groups were done with three repetitions. The fermentation culture after treatments were filtered and the obtained mycelia were blotted on filter papers. Then the mycelia were weighed and collected in a 1.5 ml centrifuge tube and frozen by liquid nitrogen immediately and stored at −80 °C for later RNA extraction and TPS activity determination. The RNA was extracted and the cDNA was synthesized as described in the former section (2.2 RNA extraction and cDNA synthesis). One gram of *P. tuoliensis* mycelia through each treatment were ground with liquid nitrogen and extracted in two ml of 50 mM PBS buffer (pH 7.0) for 10 min on ice. Then the extractions were centrifuged at 12,000 g and 4 °C for 20 min and the supernatant was used as the crude enzyme solutions to be tested for TPS activity.

### Quantitative PCR analysis

To investigate the expression of *P. tuoliensis TPS* gene under heat stress, qPCR was conducted by using SYBR^®^ FAST qPCR Kits (Kapa Biosystems, Wilmington, MA, USA) according to the manufacturer’s instructions. Beta-actin was used as a housekeeping gene and the data were calculated with the 2^−ΔΔt^ method (Livak & Schmittgen, 2001) to normalize the expression. The qPCR primers qPCR-TPS-F/R and qPCR-β-actin-F/R were listed in Table S1.

Each PCR reaction solution contained 10 μl of SYBR^®^ FAST qPCR Master Mix, 0.4 μl of Rox Low Master Mix, two μl of cDNA and 0.4 μl of each qPCR primer (10 μM), and 6.8 μl of ddH_2_O. The program was run as follows: an initial denaturation at 95 °C for 3 min, followed by 40 cycles at 95 °C for 3 s, 57 °C for 32 s, then a final extension at 72 °C for 30 s. A melting curve analysis was performed at last to confirm the effect of amplification.

### In vitro expression of TPS in *E. coli* and purification

Two primers, TPS-*Bam*HI-F and TPS-*Hin*dIII-R with *Bam*HI and *Hin*dIII restriction enzyme sites respectively, were used to amplify *P. tuoliensis TPS* cDNA fragment. The purified PCR product was double-digested with *Bam*HI and *Hin*dIII, and ligated into the predigested pET28a vector (Novagen, Inc., Madison, WI, US). The recombinant plasmid verified by DNA sequencing was designated as pET28a-TPS and was then transformed into *E. coli* BL21 (DE3) cells. The positive transformant strain was inoculated to LB medium with 50 μg/ml of kanamycin and was shaken at 37 °C, 180 rpm until its absorption at OD600 nm reached 0.6. The culture was inducted by adding isopropyl-b-D-thiogalactopyranoside (IPTG) at the final concentration of one mM and was shaken at 25 °C, 160 rpm for 3 h. The culture cells were obtained by centrifugation and washed once with PBS buffer (137 mM NaCl, 2.7 mM KCl, 10 mM Na_2_HPO_4_, and 2 mM KH_2_PO_4_, pH 7.4), and were resuspended in the binding buffer (pH 8.0, 50 mM phosphate buffer with 300 mM NaCl and 10 mM imidazole). Cells were disrupted by ultrasonication and the intracellular enzymes were kept in the supernatant after centrifugation. The crude enzyme extract was applied to Ni-NTA agarose (Qiagen, Duesseldorf, Germany) in a column which had been previously equilibrated by the binding buffer. Then the column was eluted with the binding buffer, the washing I buffer (same as binding buffer in addition with 20 mM imidazole), the washing II buffer (same as binding buffer in addition with 80 mM imidazole), and the elution I buffer (same as binding buffer in addition with 100 mM imidazole) successively. Each fraction of eluate was analyzed by SDS-PAGE. The fraction with a single band and the estimated molecular weight on the gel was tested for TPS activity. Then the verified fraction was ultrafiltrated to remove the salt and to concentrate the enzyme. The obtained concentrated solution was considered as the purified recombinant TPS and was stored at −80 °C for further assays.

### Standard TPS activity assay

The TPS activity was assayed by a two-step reaction method as described by Hottiger et al. with some modifications (Hottiger, Schmutz & Wiemken, 1987). The 200 μl of assay mixture in the first step contained 2.5 mM G-6-P, 2.5 mM UDPG, 12.5 mM MgCl_2_, 10 μl of the purified PtTPS or the crude enzyme solution, 30 mM Tris/HCl buffer (pH 7.4) and ddH_2_O. After the mixture was incubated at 30 °C for 30 min, the reaction was stopped by boiling for 5 min and putting on ice for 10 min. The reaction mixture was then centrifuged at 2,000 g for 10 min to get the supernatant, which was added to the second assay mixture containing two mM phosphoenolpyruvate (PEP), 0.3 mM NADH, 30 mM Tris/HCl (pH 7.4), five U lactic acid dehydrogenase and water. The second reaction was started by adding five U pyruvate kinase. The absorbance at OD340 nm was measured per minute until no change and the decrease was used to calculate the concentration of uridine diphosphate (UDP). The assay was run at 30 °C. One unit of the enzyme activity was defined as the amount of the enzyme generating one μmol UDP per minute.

### Assay for optimum pH and temperature

To determine the optimum pH, the TPS activity was measured at 30 °C for 30 min in several buffer systems with pH ranging from pH 3 to 7 in Na_2_HPO_4_-citric acid buffer, pH 7–9 in Tris/HCl buffer, pH 9–10 in glycine-NaOH buffer. The optimum temperature was assayed by determining the TPS activity at various temperatures (20–90 °C) at pH 7.4. The pH and temperature correspondent to maximum TPS activity were determined as the optimum pH and temperature, respectively.

### Substrate specifity

Nucleosides diphosphate glucose UDPG, ADPG, GDPG were used as glucosyl donors, respectively, and G-6-P was used as the general glucosyl acceptor in the enzyme assays. In another set, UDPG was taken as the universal glucosyl donor while G-6-P, glucosamin-6-phosphate (GS-6-P), fructose-6-phosphate (F-6-P), mannose-6-phosphate (M-6-P) were treated as glucosyl acceptors.

### Effects of metal cations on TPS activity

The activity of the purified TPS was tested as the standard method mentioned above except 12.5 mM of various metal cations instead of Mg^2+^ in the first step, respectively. The assay sample without any metal cations was set as the control.

### Effects of metal chelators and polyanions

Metal chelators such as sodium citrate, citric acid, EDTA, EGTA, and CDTA were used as additives at concentration of 2.5 mM in the assay mixture at the first step to study their effects on TPS activity. Similarly, two polyanions were added with various concentrations in the standard reaction mixtures to study their effects, respectively. The assay with none additives were regarded as the control. The activity of the control was considered as 100% and the other activities for the assay with additives were calculated as percentages of the control.

## Results

### Cloning of *P. tuoliensis TPS* cDNA

Degenerate PCR using primers TPS_F1 and TPS_R1 generated one band of 1.4 kb on an electrophoretic gel (Fig. S1). The encoding sequences of the PCR fragment shared more than 80% amino acid identities with several TPS proteins, which revealed it was a part of *TPS* gene. The 5′-RACE generated a single band of 400 bp (Fig. S2), while the 3′-RACE produced one band of 780 bp (Fig. S3). The sequences of the two RACE PCR products provided the basis for designing specific primers to clone full length of *TPS* cDNA. The amplified full cDNA fragment contains an open reading frame of 1,665 nucleotides encoding 554 amino acids.

### Sequence analysis of *P. tuoliensis TPS*

**Figure 1:**
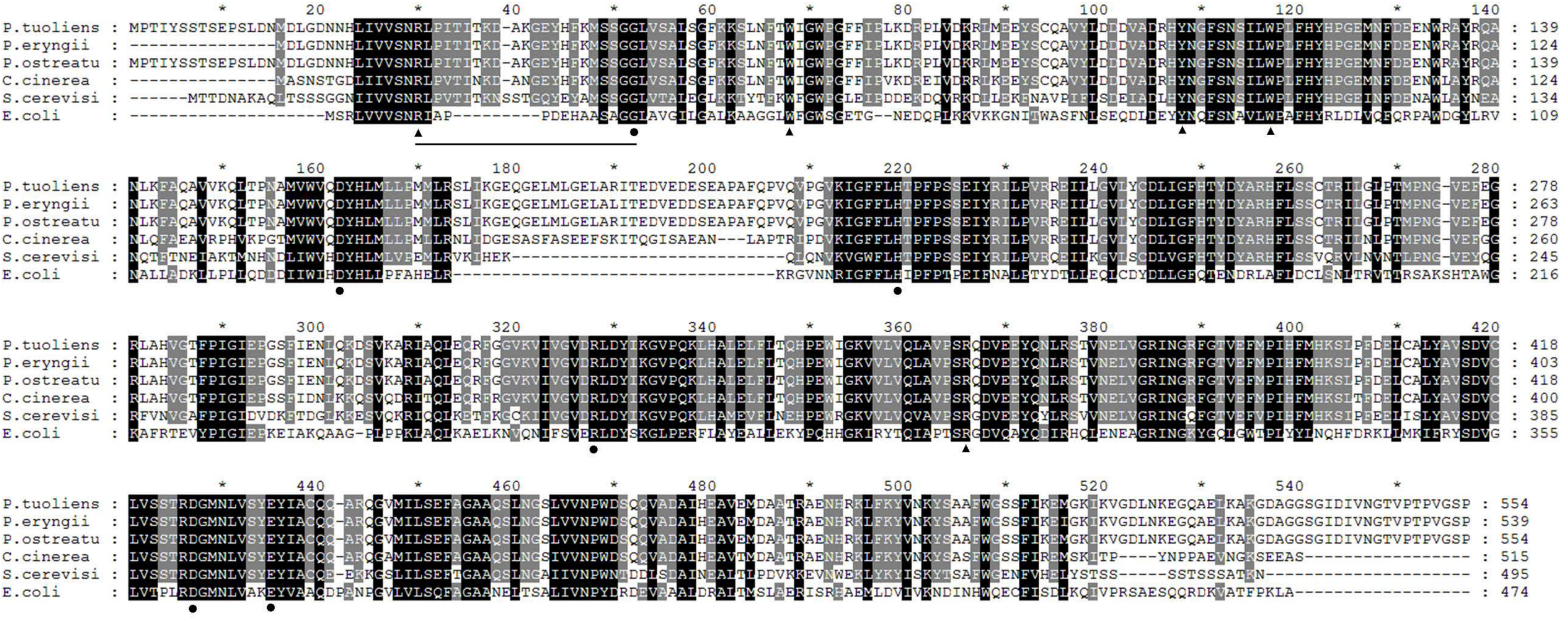
The alignment of the amino acid sequences of *P. tuoliensis* TPS with some of its homologous proteins. The alignment was performed with the ClustalX program. Invariant and highly conserved amino acids were shaded. Dashes indicated gaps introduced during the alignment. The N-loop region which interacts extensively with the glycosyl acceptor site was underlined. The conserved residues Arg30, Trp67, Tyr107, Trp116, and Arg364 involved in the binding of the glucose-6-phosphate acceptor were indicated as filled triangles, while residues Gly51, Asp161, His218, Arg326, Asp425, and Glu433 involved in the binding of UDP-glucose donor were marked as filled circles.

The amino acid sequence of *P. tuoliensis* TPS were blasted with its homologues from several other organisms (Fig. 1). It shared 96.75% identity with *P. eryngii* (jgi|Pleery1|1445745), 99.82% with *P. ostreatus* (MF673394), 77.98% with *Coprinopsis cinerea* (XP_001836488.2), 54.23% with *S. cerevisiae* (Q00764.2) and 27.52% with *E. coli* (P31677.3). *P. tuoliensis* TPS shared high sequence identities with its homologues of fungi, in especial macro fungi. Our alignment also demonstrated some catalysis-related conserved amino acids. The residues Arg30, Trp67, Tyr107, Trp116, and Arg364 are involved in the binding of the glucosyl acceptor, and the residues Gly51, Asp161, His218, Arg326, Asp425, and Glu433 are involved in the binding of glucosyl donor. Therefore, the cDNA sequence was named *PtTPS* and could be obtained with the accession number MF674013 at GenBank.

### Relative expression of *TPS* gene after heat stress

**Figure 2:**
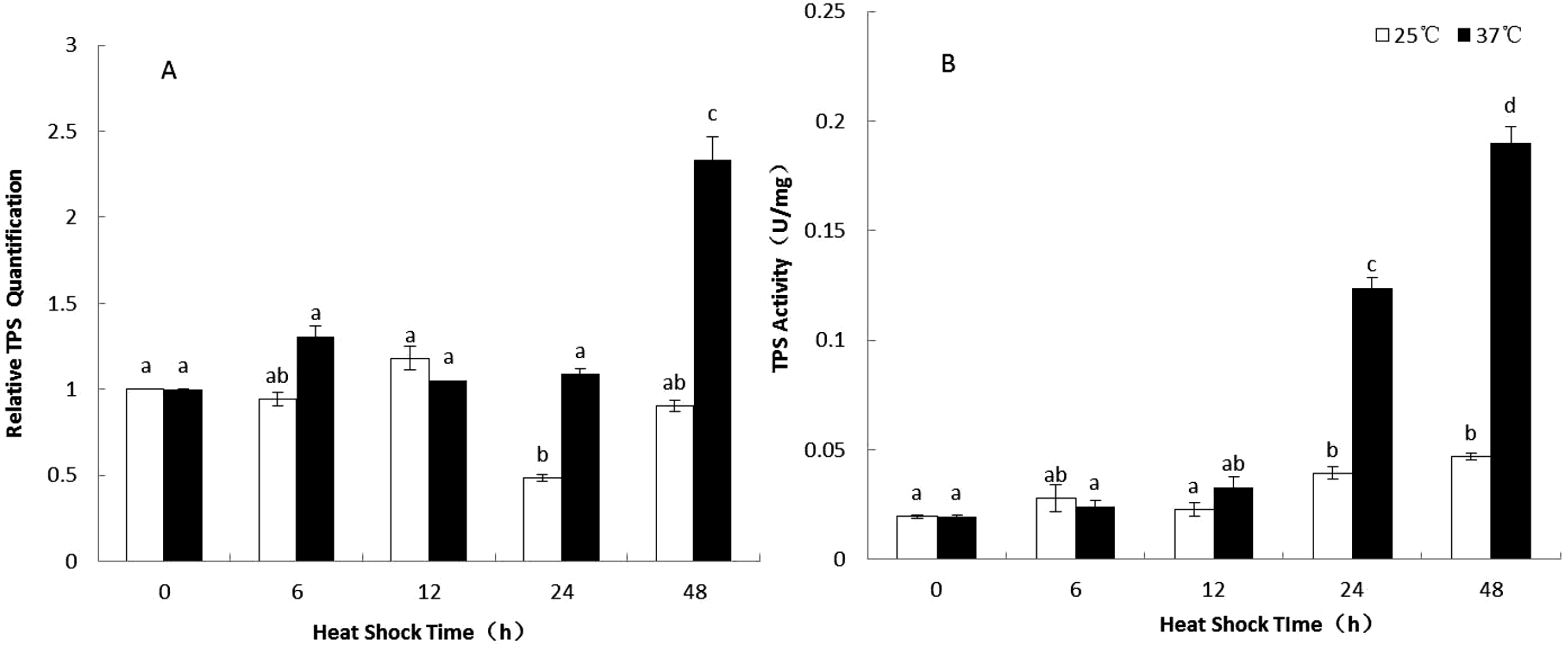
The relative quantification of *TPS* gene (A) and the enzyme activity of TPS in *P. tuoliensis* (B). White bars were for 25 °C and black bars were for 37 °C (*P* ≤ 0.05 according to Duncan’s multiple range test).

The relative expression levels of *PtTPS* gene significantly increased during 24–48 h of heat stress treatment at 37 °C compared to those at 25 °C while no significant changes were detected during 0–12 h. (Fig. 2A). The activity of TPS showed a similar trend as that of the expression levels (Fig. 2B). These results revealed that the heat stress induced both *PtTPS* gene expression and TPS enzyme activity during later stage (24–48 h) of heat stress (Figs. 2A and 2B).

### In vitro expression of TPS and purification

**Figure 3:**
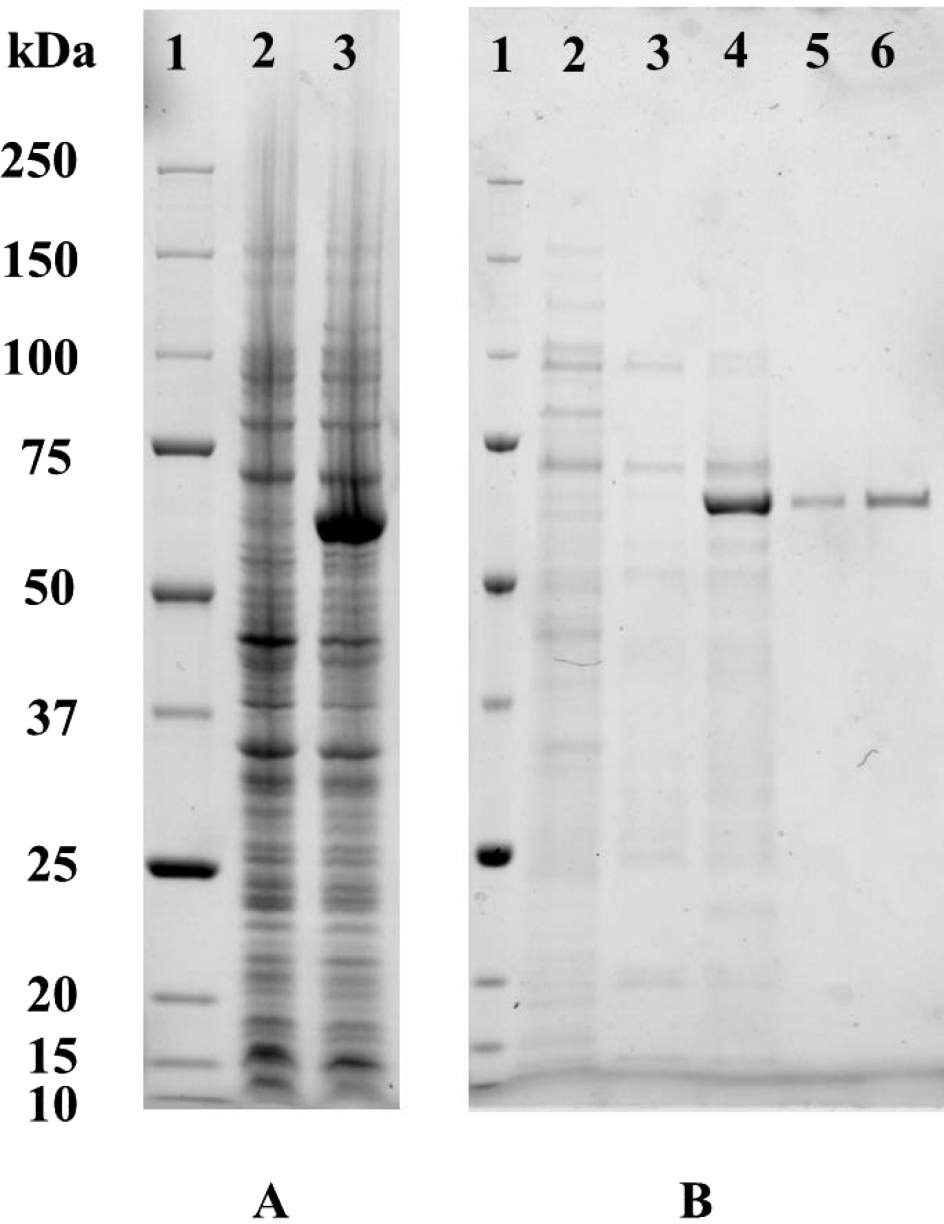
In vitro over-expression and purification of the recombinant *P. tuoliensis* TPS protein. (A) Over-expression of TPS protein. Lane 1: Biorad unstained protein standard (CAT#: 161-0363); lane 2: total lysate before IPTG induction; lane 3: total lysate after IPTG induction. (B) Purification of TPS protein at Ni-NTA. Lane 1: Biorad unstained protein standard (CAT#: 161-0363); lane 2: the eluate by the binding buffer; lane 3: the eluate by the washing I buffer; lane 4: the eluate by the washing II buffer; lane 5: the eluate by the elution I buffer; lane 6: ultrafiltrate of the eluate by the elution I buffer.

The expression and purification of PtTPS were analyzed by SDS-PAGE as shown in Fig. 3. The recombinant TPS under IPTG induction was expressed at high level in *E. coli* (Fig. 3A). The TPS was purified by affinity chromatography through Ni-NTA (Ni^2+^-nitrilotriacetic acid) column and several buffer solutions were used to elute the column. A lot of proteins were eluted by the binding buffer, the washing I buffer and washing II buffer. Although a large amount of the interested protein were eluted from the column by the washing II buffer, a number of other proteins also existed in the eluted solutions. The pure TPS protein with high TPS activity was eluted by the elution I buffer. It appeared in the gel with a molecular mass of about 60 kDa identical with the theoretical molecular mass (Fig. 3B). Thus the eluent by the elution I buffer was ultrafiltrated. The concentrated solution was considered as purified TPS protein and was used for further test.

### The optimum temperature and pH

**Figure 4:**
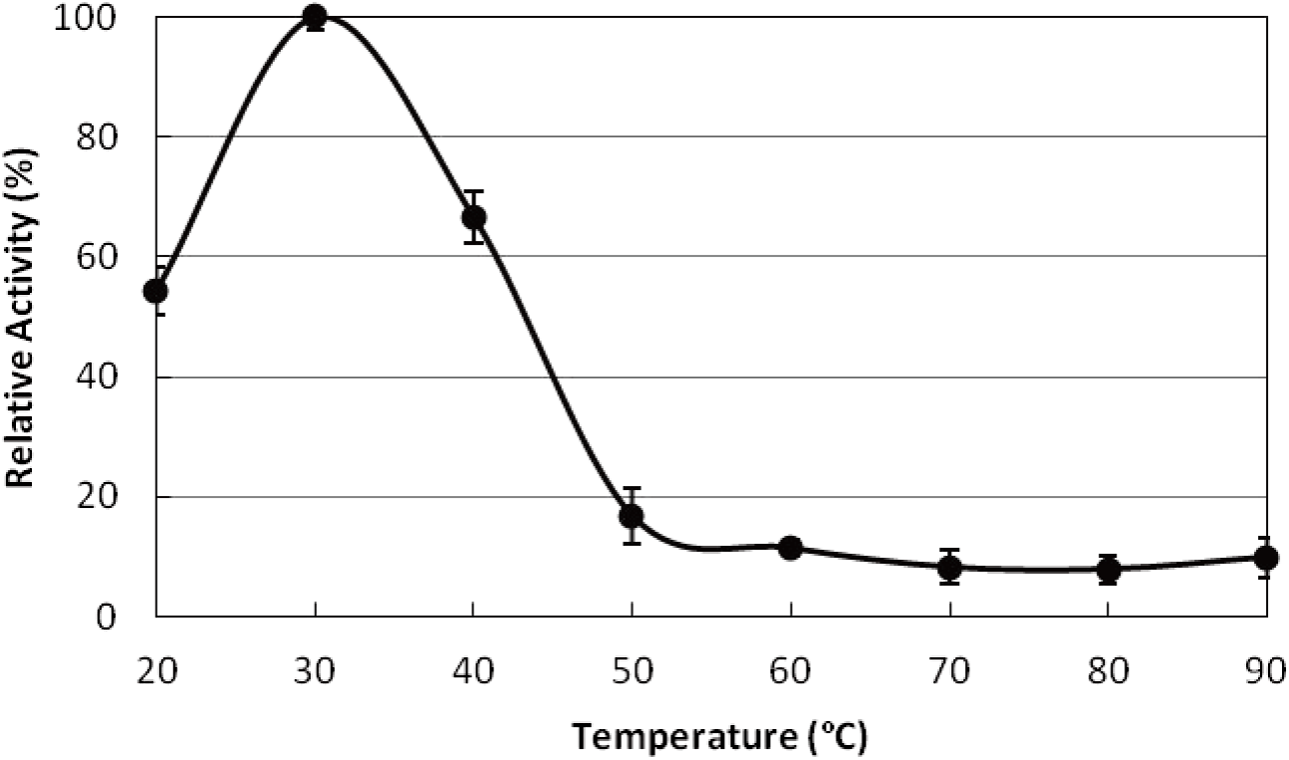
The optimum temperature for TPS activity. The activity at 30 °C was taken as 100%. All the data came from three independent experiments. Error bars indicated the corresponding standard deviations.

**Figure 5:**
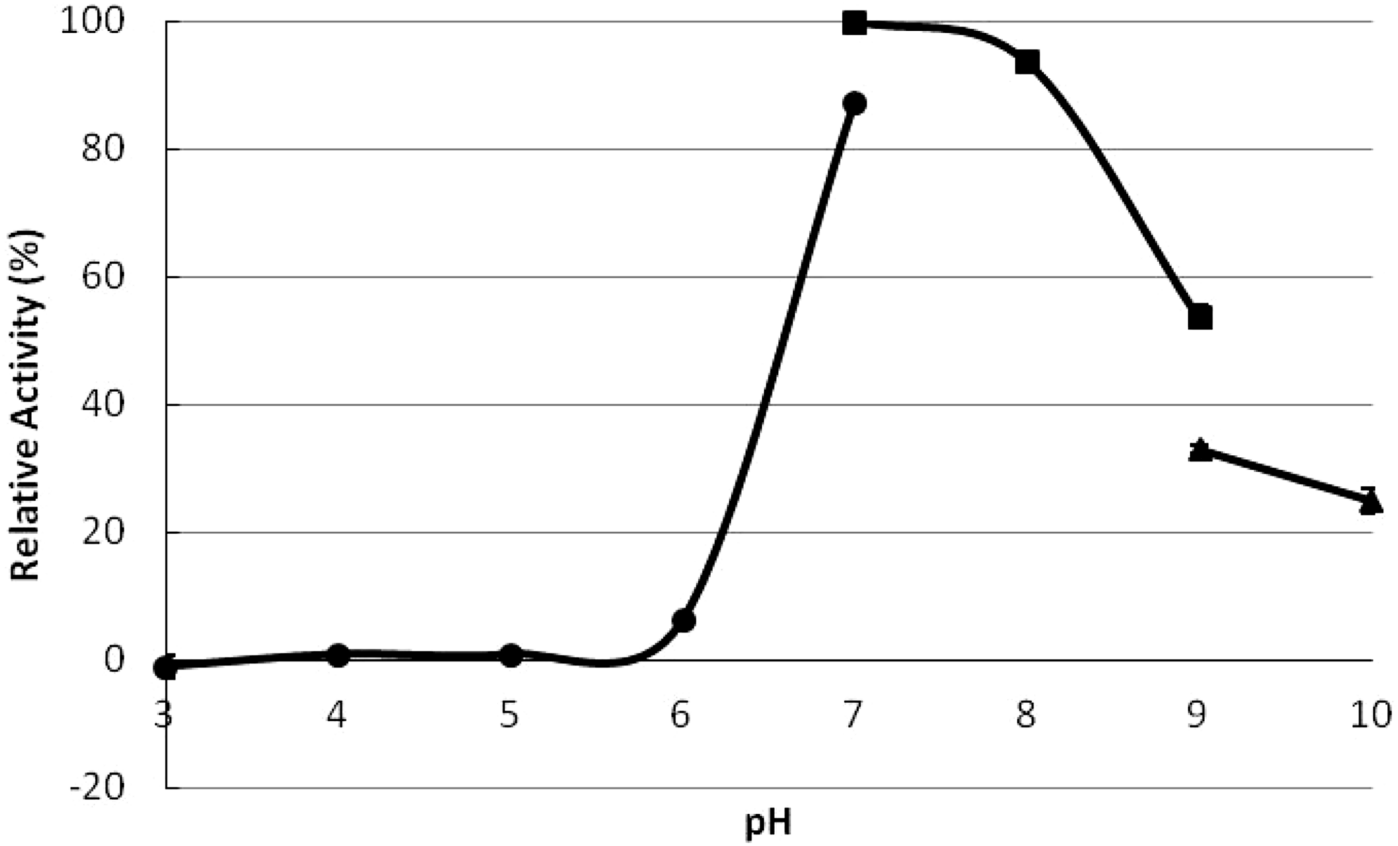
The optimum pH for TPS activity. The activity at pH 7 was taken as 100%. Circles denoted the Na_2_HPO_4_-citric acid buffer, squares stand for Tris/HCl buffer and triangles indicated the glycine-NaOH buffer. All the data came from three independent experiments. Error bars indicated the corresponding standard deviations.

The optimum temperature for the *P. tuoliensis* TPS activity was 30 °C (Fig. 4). The TPS activity retained more than half of the maximum both at 20 °C and 40 °C, while it declined abruptly as the temperature climbed to 50 °C and kept constantly around 10% of the maximum until 90 °C. The activity of TPS was severely affected at low pH value (below pH 6) and reached the maximum at pH 7 (Fig. 5). The enzyme acted more efficiently in neutral to alkaline conditions as more than 50% activity exists at pH 7–9 in Tris/HCl buffer.

### Substrate specificity

**Figure 6:**
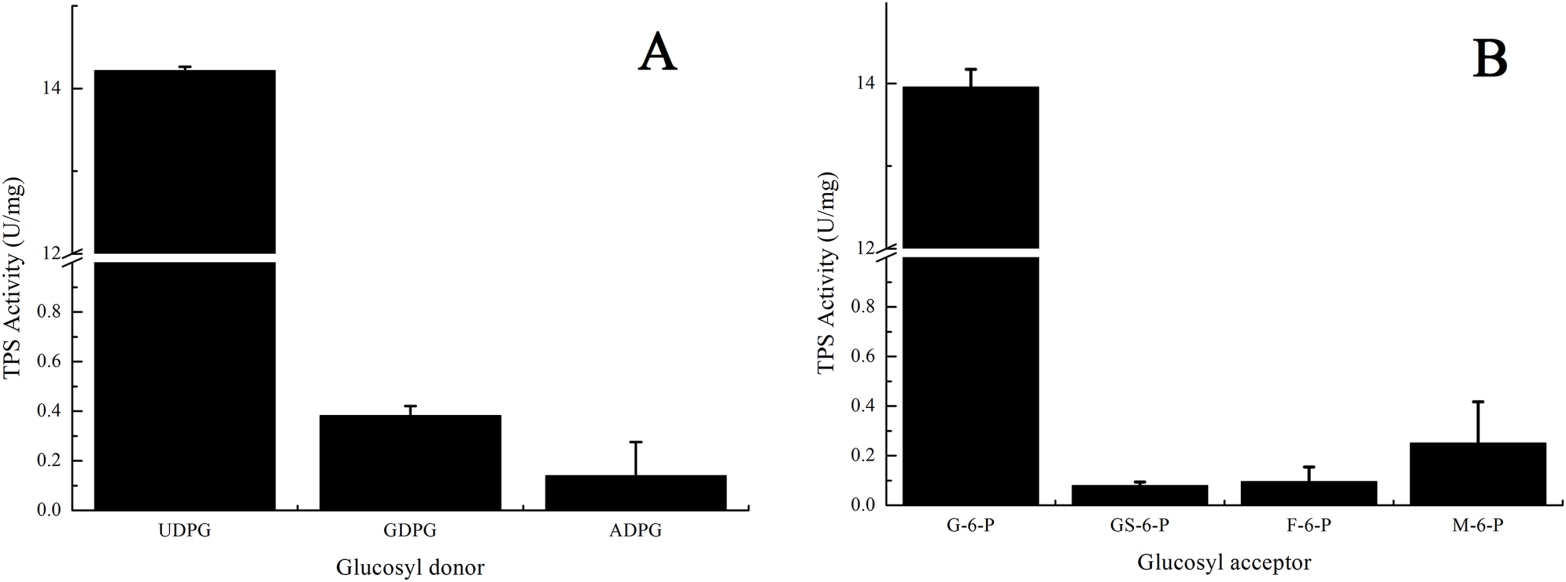
Substrate specificity of PtTPS. (A) Specificity of glucosyl donor. (B) Specificity of glucosyl acceptors. All the data came from three independent experiments. Error bars indicated the corresponding standard deviations.

Three glucosyl donors were used to test their potential roles as substrates for the enzyme TPS. UDPG was the best glucosyl donor for TPS protein among the three and the TPS activity with UDPG was 14.22 U/mg (Fig. 6A). Both guanosine diphosphate glucose (GDPG) and adenosine diphospate glucose (ADPG) gave little TPS activity, 0.38 U/mg and 0.14 U/mg respectively (Fig. 6A). G-6-P was the only appropriate one among the four tested glucosyl acceptors. GS-6-P, F-6-P showed almost no TPS activity, while M-6-P exhibited little activity (Fig. 6B).

### Effects of metal cations and chelators on the TPS activity

The PtTPS enzyme required some cations to function, as its activity without additions of any metal cations was only 0.163 U/mg (Table 1). Supplement of Mg^2+^, Co^2+^, Mn^2+^, Ni^2+^, K^+^, and Ag^+^, respectively, significantly stimulated the TPS activity. The Mg^2+^ was the most appropriate cations to activate TPS activity as it increased the TPS activity by more than 7,000 fold. No tested metal cations had significant inhibitive effects on TPS activity (Table 1). Various metal chelators were applied to test their effects on TPS activity. All the tested chelators except for the sodium citrate inhibited the TPS activity seriously (Table 2).

**Table 1:** Effect of metal cations on the TPS activity.

Metal	Concentration (mM)	Specific activity (U/mg)	Activity (%)
None	–	0.163 ± 0.04^f,g,h^	100
Mg^2+^	12.5	12.797 ± 0.12^a^	7,851.20
Co^2+^	12.5	3.133 ± 0.05^b^	1,922.04
Mn^2+^	12.5	0.775 ± 0.11^c^	475.71
Ni^2+^	12.5	0.657 ± 0.13^c^	402.82
K^+^	12.5	0.469 ± 0.10^c^	287.73
Ag^+^	12.5	0.363 ± 0.02^d,e^	222.51
Zn^2+^	12.5	0.266 ± 0.03^e,f^	163.05
Fe^2+^	12.5	0.263 ± 0.16^e,f^	161.13
Cu^2+^	12.5	0.203 ± 0.10^f,g^	124.68
Ca^2+^	12.5	0.066 ± 0.02^g,h^	40.28
Cd^2+^	12.5	0.066 ± 0.03^g,h^	40.28
Li^+^	12.5	0.059 ± 0.01^g,h^	36.45
Fe^3+^	12.5	0.056 ± 0.03^g,h^	34.53
Na^+^	12.5	0.034 ± 0.03^h^	21.10

**Note:**

All the experiments were performed in triplicate. All the experiments were done three times and the values indicate the mean ± SD. The different superscript letters indicate a significant difference. (*P* ≤ 0.05 according to Duncan’s multiple range test).

**Table 2:** Effects of metal chelators on PtTPS activity.

Chelators	Concentration (mM)	Specific activity (U/mg)	Residual activity (%)
None	2.5	11.995 ± 0.07^a^	100
Sodium citrate	2.5	11.273 ± 0.09^b^	93.98
Citric acid	2.5	0.424 ± 0.20^c^	3.55
EDTA	2.5	0.052 ± 0.03^d^	0.43
EGTA	2.5	0.026 ± 0.04^d^	0.22
CDTA	2.5	0.010 ± 0.02^d^	0.08

**Note:**

TPS activity was assayed as introduced in the methods. All the experiments were done three times and the values indicate the mean ± SD. The different superscript letters indicate a significant difference. (*P* ≤ 0.05 according to Duncan’s multiple range test).

### Regulation of the TPS activity by polyanions

**Figure 7:**
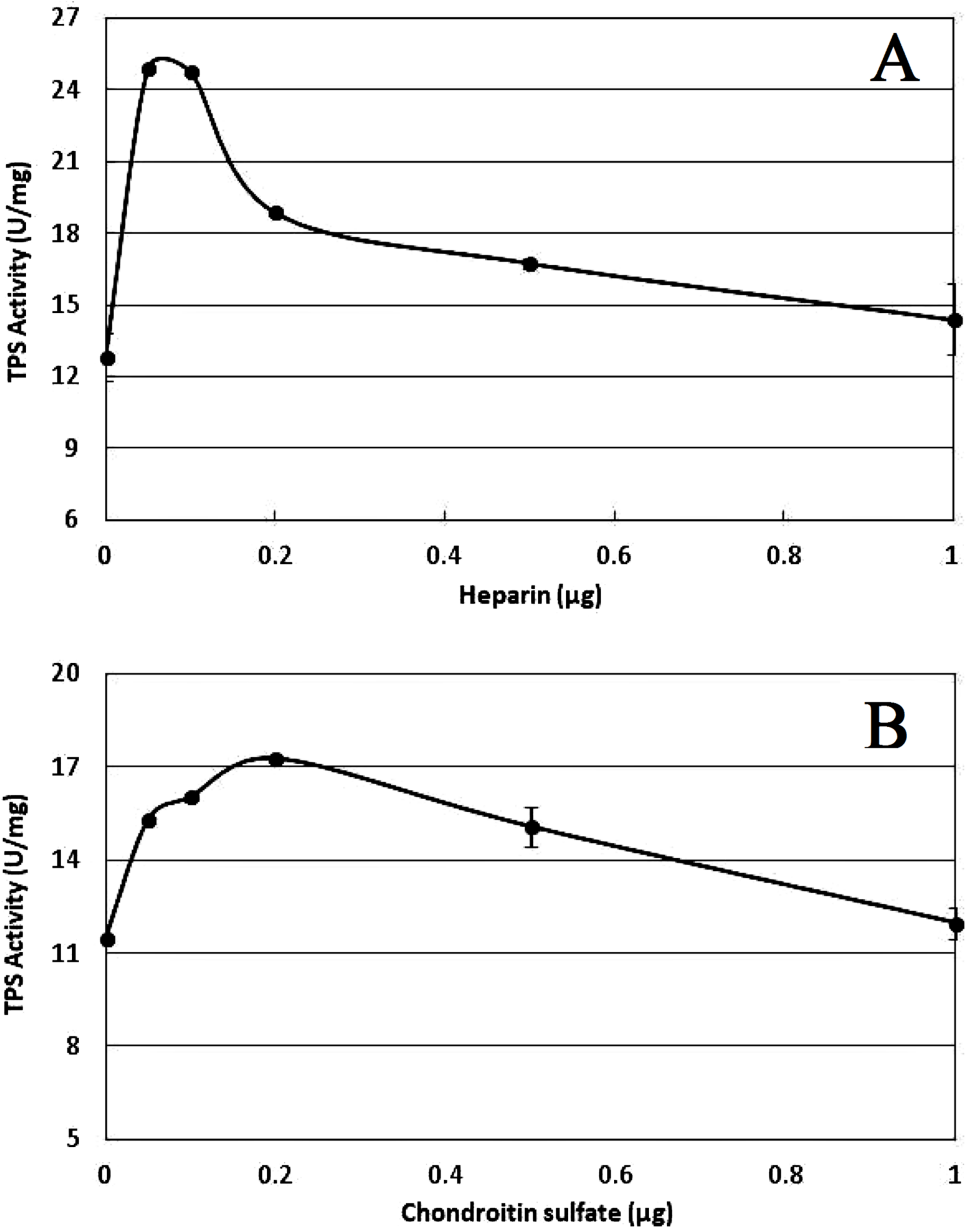
Effects of polyanions on PtTPS activity. (A) Heparin. (B) Chondroitin sulfate. All the data came from three independent experiments. Error bars indicate the corresponding standard deviations.

The polyanions heparin and chondroitin sulfate were added at various amounts to test their effects on TPS activity. Both heparin and chondroitin sulfate, supplemented at the amount ranging from 0.1 to 0.5 μg, greatly stimulated the TPS activity. The TPS activity increased abruptly at the addition of 0.1 μg heparin, while it decreased rapidly at the addition of 0.2 μg heparin (Fig. 7A). The TPS activity in the supplement of chondroitin sulfate rose and climbed to the maximum at the addition of 0.2 μg chondroitin sulfate, then decreased gradually (Fig. 7B).

## Discussions

Trehalose has been demonstrated as a stress protectant in many organisms. The main biosynthetic pathway TPS-TPP was found firstly and studied extensively. A lot of researches on the protective role of *TPS* gene to various environmental stresses including heat stress have been reported in the last few decades. In *S. cerevisiae*, *TPS* (*TPS1*) mutation suppressed the accumulation of trehalose after heat shock and significantly lowered the heat-induced thermotolerance (De Virgilio et al., 1994). In *A. nidulans*, *TPS* (*tpsA*) mutant behaved more sensitively to heat stress (Fillinger et al., 2001). In our previous study with *P. tuoliensis*, the transcription of TPS gene was induced after heat stress and the addition of the trehalose alleviated the damage caused by heat stress (Kong et al., 2012).

Due to the important roles of TPS, many groups purified the TPS proteins and characterized their properties from several organisms, such as *S. cerevisiae* (Chaudhuri, Basu & Ghosh, 2008), *Selaginella lepidophylla* (Valenzuela-Soto et al., 2004), *Thermus thermophilus RQ-1* (Silva, Alarico & da Costa, 2005), *Metarhizium anisopliae* (Cai et al., 2009), *Candida utilis* (Sengupta et al., 2011), *Thermoplasma acidophilum* (Gao et al., 2014), *P. ostreatus* (Lei et al., 2017), and *R. oryzae* (Uyar, Yucel & Hamamci, 2016). The protein structure of some TPS proteins from *E. coli* (Gibson et al., 2002, 2004), *C. albicans* (Miao et al., 2017), and *A. fumigatus* (Miao et al., 2017) have been elucidated and the structure-related catalytic mechanisms were thus inferred.

In this study, the full length of TPS cDNA was cloned from *P. tuoliensis*. Homologous analysis of PtTPS amino acids showed high homology with other fungi, especially the macro fungi (Fig. 1). High homologies of TPS amino acids were also shown among algal species (Deng et al., 2014), filamentous fungi (Cai et al., 2009), and diverse insect species (Kern et al., 2012), respectively. It was reported in *E. coli* that a deep fissure existed at the interface of two structural domains to form the *TPS* (OtsA) catalytic center (Gibson et al., 2002). The alignment of TPS proteins from insects, bacteria, yeast, fungi, nematodes, and plants also showed that the TPS proteins have two conserved motifs (Tang et al., 2010). Most of the highly conserved regions were all involved in the substrate binding and catalysis (Kosmas et al., 2006). Similarly in this study, PtTPS was also highly conservative in these regions (Fig. 1). The conserved N-loop region from Arg30 to Gly51 was reported to interact with glucosyl donor and acceptor domains (Gibson et al., 2004) and was relevant to the catalytic efficiency of OtsA (Jiang et al., 2010).

The relative expression of *PtTPS* and enzyme activity were induced significantly after heat stress (Fig. 2). It revealed that the TPS responded actively to heat stress, which is a common characteristic of TPS in many researches (Bell et al., 1992; Hottiger, Schmutz & Wiemken, 1987; Lei et al., 2017). Compared with our previous study with *P. ostreatus*, PtTPS responded actively at the later stage of heat stress while the TPS from *P. ostreatus* responded actively at the early stage of heat stress (Lei et al., 2017). The induced *TPS* may stimulate the bio-synthesis of trehalose (Doehlemann, Berndt & Hahn, 2006; Miranda et al., 2007; Reina-Bueno et al., 2012; Soto et al., 1999) or the product of TPS protein itself (Gibney et al., 2015; Petitjean et al., 2015) which might help the cells to tolerate the heat stress.

The optimum temperature of PtTPS was 30 °C, the same as that of the TPS in *P. ostreatus* (Lei et al., 2017). Most reported TPS exhibited highest activities between 30 and 40 °C such as 35 °C *in Ascaris suum* (Dmitryjuk et al., 2013), 37 °C in *C. utilis* (Sengupta et al., 2011), 40 °C in *S. cerevisiae* (Chaudhuri et al., 2009), and *M. anisopliae* (Cai et al., 2009). Some thermophilic species showed even higher optimum temperature, such as 60 °C in *T. acidophilum* (Gao et al., 2014) and extremely as high as 98 °C in *T. thermophilus RQ-1* (Silva, Alarico & da Costa, 2005). The optimum pH of most previously reported TPS was around the neutral pH from 6 to 8.5, such as 6 in *T. acidophilum* (Gao et al., 2014) and *T. thermophilus RQ-1* (Silva, Alarico & da Costa, 2005), 6.5 in *M. anisopliae* (Cai et al., 2009), 7 in *S. lepidophylla* (Valenzuela-Soto et al., 2004), 7.4 in *P. ostreatus* (Lei et al., 2017), 8.5 in *S. cerevisiae* (Chaudhuri et al., 2009), and *C. utilis*(Sengupta et al., 2011). The optimum pH of PtTPS was 7 (Fig. 5), which is as same as TPS of *S. lepidophylla* (Valenzuela-Soto et al., 2004) and very close to *P. ostreatus* (7.4) (Lei et al., 2017), but PtTPS was active at a range of alkaline pH while the latter two are both more active in acidic buffer.

This study showed that UDPG and G-6-P had maximum activities among all the listed glucosyl donors and acceptors (Fig. 6). It was as same as the TPS in *Mycobacterium smegmatis* (Lapp, Patterson & Elbein, 1971), *Dictyostelium discoideum* (Killick, 1979), *Mycobacterium tuberculosis* (Pan, Carroll & Elbein, 2002), *Euglena gracilis* (Fiol & Salerno, 2005), *S. cerevisiae* (Chaudhuri et al., 2009), and *T. acidophilum* (Gao et al., 2014). It was thus proved that UDPG and G-6-P were the most suitable TPS substrates. The glucosyl donor GDPG showed only 2.8% of the activity with the optimum donor UDPG in our study (Fig. 6), much lower than 50% of that in *S. cerevisiae* (Chaudhuri et al., 2009) and 20% of that in *T. acidophilum* (Gao et al., 2014). The glucosyl acceptor M-6-P showed only 2% of the activity with the optimum acceptor G-6-P in our study (Fig. 6), also much lower than 90% of that in *S. cerevisiae* (Chaudhuri et al., 2009) and 40% of that in *T. acidophilum* (Gao et al., 2014). Thus, it revealed that PtTPS possessed high substrate specificity of glucosyl donors and acceptors.

The PtTPS activity was very low without the addition of any metal cations (Table 1). The Mg^2+^ and Co^2+^ were the best two activators as they stimulated the TPS activity tremendously (Table 1). The Mg^2+^ was a universal stimulator for TPS in many previous reports. The K^+^ in *D. discoideum* (Killick, 1979), Zn^2+^ in *S. cerevisiae* (Chaudhuri et al., 2009), and Zn^2+^, Co^2+^, Mn^2+^ in *T. acidophilum* (Gao et al., 2014) also acted as stimulators of TPS, which were the same case in our results (Table 1). In addition, all the tested metal chelators (including specific and non-specific metal chelators) inhibited PtTPS activity significantly (Table 2). As a result, it seemed that the metal cations were mandatory for PtTPS activity.

A certain concentration of polyanions like heparin and chondroitin sulfate stimulated the activity of TPS according to most previously reported TPS, such as TPS in *M. smegmatis* (Lapp, Patterson & Elbein, 1971), *D. discoideum* (Killick, 1979), *M. tuberculosis* (Pan, Carroll & Elbein, 2002), *S. cerevisiae* (Chaudhuri et al., 2009), *C. utilis* (Sengupta et al., 2011), and *T. acidophilum* (Gao et al., 2014). The stimulatory effects of the polyanions on TPS in *P. tuoliensis* were also dose-dependent. The molecular mechanisms between the protein and the polyanions are not well understood, but the presence of conserved amino acids regions with relation to enzyme activation and the polyanion had been discovered by amino acid analysis of the TPS molecule (Chaudhuri, Basu & Ghosh, 2008).

## Conclusions

Overall, we cloned the full length of TPS cDNA from the edible mushroom *P. tuoliensis* and found the TPS reacted positively during heat stress. Then we expressed and purified the TPS from *E. coli* and characterized the biochemical properties of recombinant TPS. The PtTPS showed high identities in the conserved catalytic domains and the similar molecular mass, optimum temperature, and pH with the other most discovered analogs. However, the PtTPS showed high substrate specificity and strong dependency of metal cations. To our knowledge, this is the first report about the characterization of TPS from *P. tuoliensis*. The study offered some basic information for further research on the trehalose metabolisms in the heat stress responses of fungi.

## Supplemental Information

10.7717/peerj.5230/supp-1 Supplemental Information 1 Table S1. The primers used in the study. Note: The restriction enzyme sites were underlined.

10.7717/peerj.5230/supp-2 Supplemental Information 2 Fig. S1. Degenerate PCR using primers TPS_F1 and TPS_R1. Lane 1: Tiangen D2000 DNA Marker, Lane 2: Degenerate PCR product.

10.7717/peerj.5230/supp-3 Supplemental Information 3 Fig. S2. 5’RACE PCR. Lane 1: Tiangen DNA Marker III, Lane2: 5’ RACE PCR product.

10.7717/peerj.5230/supp-4 Supplemental Information 4 Fig. S3. 3’RACE PCR. Lane 1: Tiangen DNA Marker III, Lane2: 3’ RACE PCR product.

10.7717/peerj.5230/supp-5 Supplemental Information 5 Dataset S1. The cDNA sequence of PtTPS.

10.7717/peerj.5230/supp-6 Supplemental Information 6 Raw data.

## Additional Information and Declarations

### DNA Deposition

The following information was supplied regarding the deposition of DNA sequences: The cDNA sequence was named PtTPS and is available at GenBank (accession number MF674013).

### Data Availability

The following information was supplied regarding data availability: The primers used in this study are listed in Table S1. The cDNA sequence is presented in Dataset S1.
