# Supplementary material for: Cloning, purification and characterization of trehalose-6-phosphate synthase from Pleurotus tuoliensis
Source: PeerJ. 2018 Jul 12;6:e5230. doi: 10.7717/peerj.5230 (PMC6046196; doi:10.7717/peerj.5230)
Supplement: Supplemental Information 1 — Note: The restriction enzyme sites were underlined. [file peerj-06-5230-s001.docx]

| **Primer name** | **Sequence（5’-3’）** |
| --- | --- |
| TPS_F1 | CGGCTGGCCTGGCTTYTTYATHCC |
| TPS_R1 | GATAGCGTCAGCGACTTGTTGNSWRTCCCA |
| 5’RACE-TPS-GSP | GGCGTTGGGCGTCAGTTGTTTCAC |
| 5’RACE-TPS-NGSP | GGAGAAACCGTTGTAATGGCGATCTGCT |
| 3’RACE-TPS-GSP | CACGGTGAACGAATTAGTGGGGCG |
| 3’RACE-TPS-NGSP | GTGGGGCGTATCAACGGACGATTC |
| TPS-F2 | CCCTCAATCGCCAGTCGCCC |
| TPS-R2 | CTGTCCCCCCAAACATCGCA |
| TPS-*BamH*I-F | CGGGATCCATGCCGACAATCTATTCTTCAACC |
| TPS-*Hind*III-R | CCCAAGCTTTGTCCCCCCAAACATCGCAC |
| qPCR-TPS-F | ATCACGGAAGATGTCGAAGA |
| qPCR-TPS-R | CGGCGAACAGGCCAGAAT |
| qPCR-β-actin-F | GCGATGAACAATAGCAGGG |
| qPCR-β-actin-R | GCTGGTATCCACGAGACAAC |
