# Supplementary figures and images for: Cloning, purification and characterization of trehalose-6-phosphate synthase from Pleurotus tuoliensis
Source: PeerJ. 2018 Jul 12;6:e5230. doi: 10.7717/peerj.5230 (PMC6046196; doi:10.7717/peerj.5230)

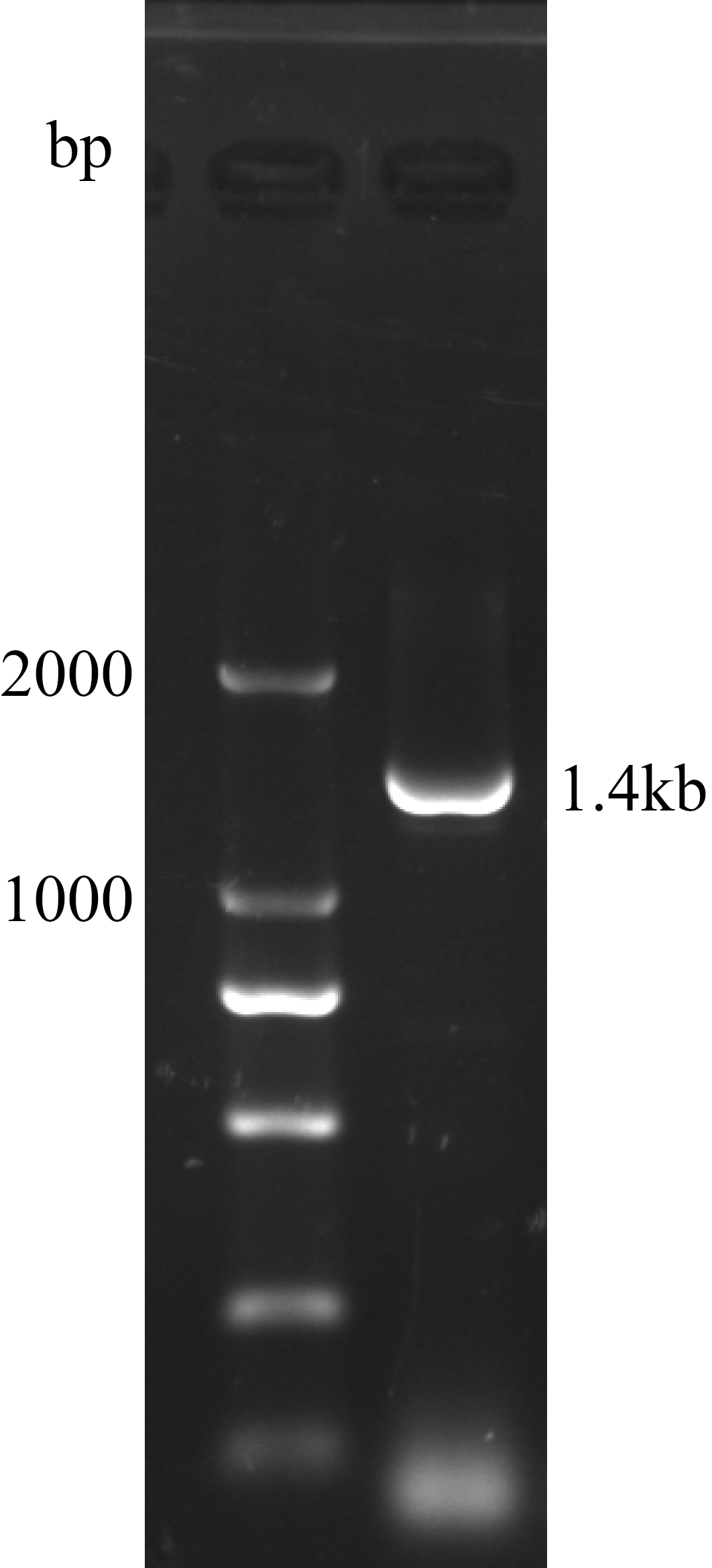

Supplement: Supplemental Information 2 — Lane 1: Tiangen D2000 DNA Marker, Lane 2: Degenerate PCR product. [file peerj-06-5230-s002.png]

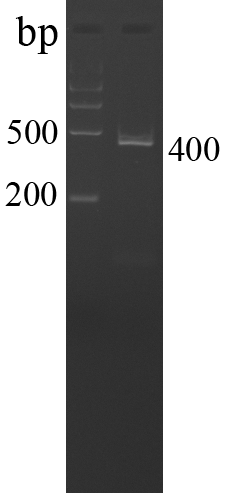

Supplement: Supplemental Information 3 — Lane 1: Tiangen DNA Marker III, Lane2: 5’ RACE PCR product. [file peerj-06-5230-s003.png]

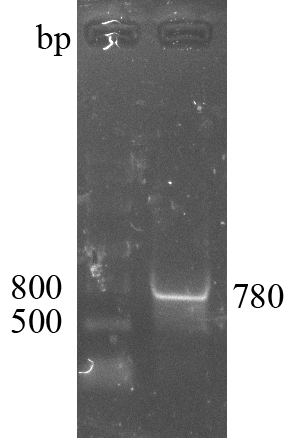

Supplement: Supplemental Information 4 — Lane 1: Tiangen DNA Marker III, Lane2: 3’ RACE PCR product. [file peerj-06-5230-s004.png]
